# Differential Expression of Serum Exosome microRNAs and Cytokines in Influenza A and B Patients Collected in the 2016 and 2017 Influenza Seasons

**DOI:** 10.3390/pathogens10020149

**Published:** 2021-02-02

**Authors:** Sreekumar Othumpangat, William G. Lindsley, Donald H. Beezhold, Michael L. Kashon, Carmen N. Burrell, Samira Mubareka, John D. Noti

**Affiliations:** 1 Allergy and Clinical Immunology Branch, Health Effects Laboratory Division, National Institute for Occupational Safety and Health, Centers for Disease Control and Prevention, Morgantown, WV 26505, USA; wdl7@cdc.gov (W.G.L.); zec1@cdc.gov (D.H.B.); ivr2@cdc.gov (J.D.N.); 2 Department of Biostatistics, Health Effects Laboratory Division, National Institute for Occupational Safety and Health, Centers for Disease Control and Prevention, Morgantown, WV 26505, USA; mqk1@cdc.gov; 3 Department of Emergency Medicine, West Virginia University, Morgantown, WV 26506, USA; cburrell@hsc.wvu.edu; 4 Department of Family Medicine, West Virginia University, Morgantown, WV 26506, USA; 5 Department of Microbiology, Division of Infectious Diseases, University of Toronto, Toronto, ON M4N 3M5, Canada; Samira.Mubareka@sunnybrook.ca

**Keywords:** exosome, microRNA, serum, cytokines, influenza virus

## Abstract

MicroRNAs (miRNAs) have remarkable stability and are key regulators of mRNA transcripts for several essential proteins required for the survival of cells and replication of the virus. Exosomes are thought to play an essential role in intercellular communications by transporting proteins and miRNAs, making them ideal in the search for biomarkers. Evidence suggests that miRNAs are involved in the regulation of influenza virus replication in many cell types. During the 2016 and 2017 influenza season, we collected blood samples from 54 patients infected with influenza and from 30 healthy volunteers to identify the potential role of circulating serum miRNAs and cytokines in influenza infection. Data comparing the exosomal miRNAs in patients with influenza B to healthy volunteers showed 76 miRNAs that were differentially expressed (*p* < 0.05). In contrast, 26 miRNAs were differentially expressed between patients with influenza A (*p* < 0.05) and the controls. Of these miRNAs, 11 were commonly expressed in both the influenza A and B patients. Interferon (IFN)-inducing protein 10 (IP-10), which is involved in IFN synthesis during influenza infection, showed the highest level of expression in both influenza A and B patients. Influenza A patients showed increased expression of IFNα, GM-CSF, interleukin (IL)-13, IL-17A, IL-1β, IL-6 and TNFα, while influenza B induced increased levels of EGF, G-CSF, IL-1α, MIP-1α, and TNF-β. In addition, hsa-miR-326, hsa-miR-15b-5p, hsa-miR-885, hsa-miR-122-5p, hsa-miR-133a-3p, and hsa-miR-150-5p showed high correlations to IL-6, IL-15, IL-17A, IL-1β, and monocyte chemoattractant protein-1 (MCP-1) with both strains of influenza. Next-generation sequencing studies of H1N1-infected human lung small airway epithelial cells also showed similar pattern of expression of miR-375-5p, miR-143-3p, 199a-3p, and miR-199a-5p compared to influenza A patients. In summary, this study provides insights into the miRNA profiling in both influenza A and B virus in circulation and a novel approach to identify the early infections through a combination of cytokines and miRNA expression.

## 1. Introduction

Influenza viruses belong to the Orthomyxoviridae family of human respiratory pathogens. In the United States, the annual direct costs of influenza illness are estimated at $4.6 billion along with more than 100 million lost workdays [1]. There are four types of influenza viruses, namely, A, B, C, and D. In recent years, influenza A and B viruses are the most commonly reported influenza viruses causing illness in humans. Influenza C infections cause mild illness and are not thought to cause infection in humans. Influenza D viruses primarily infect cattle and are not human pathogens. Influenza B virus infection is less common than infection with influenza A viruses, but studies have shown that influenza B infection can be severe in children [2]. In recent years, influenza B infection has become more common. For example, the Centers for Disease Control and Prevention, USA (CDC), tested 198 patients with influenza and found 83 cases of influenza B, 81 (98%) of which belonged to the influenza B/Victoria V1A.3 subclade, which was circulating in the United States in the latter half of the 2018–2019 influenza season [3]. Influenza viruses use the RNA-dependent RNA polymerase complex of influenza A, a trimeric protein complex encoded by genomic segments PB1, PB2, and PA, and which lacks the proof-reading ability that could lead to genetic variants in every replication cycle and escape from the selection pressure of the vaccination. In addition, the survival of the virus is host-dependent, and host cellular factors, either host-induced or virus-induced, are essential for virus survival and replication. MicroRNAs (miRNAs) are small regulatory non-coding RNAs of around 22 nucleotides in length that play a significant role in the pathophysiology of infectious viral diseases. MiRNAs are well-studied regulators of gene expression, and their up- or downregulation is a mechanism to start or shut down protein synthesis. Our laboratory [4,5,6] and others [7,8] have shown that the early stage of influenza virus infection significantly alters the miRNA profile in human lung epithelial cells. In silico analyses clearly indicate that most human mRNAs are regulated by miRNAs [9,10]. miRNAs exert their effects on the cells in which they are synthesized, and then are released into the extracellular space (through exosomes) and transported in body fluids [11,12]. Isolation of exosomal miRNA increases the sensitivity of miRNA detection as these tend to accumulate in the vesicles while transported as “cargo”. Once released into the extracellular fluid, the exosomes transfer their cargo to the acceptor cell [12,13]. miRNAs are useful for biomarker studies because they are actively sorted into vesicles, are very stable in blood plasma, serum, urine, saliva, and semen, and are easily isolated [14,15,16,17]. Moreover, exosomal miRNA signatures are altered under specific conditions and diseases, such as viral infections [18]. For example, the levels of let-7f, hsa-miR-20b, and hsa-miR-30e-3p were altered in the plasma of patients with small-cell lung cancer [19]. In addition, the exosomal hsa-miR-145 and hsa-miR-200c have been found to be altered in ovarian cancer [20]. These studies along with several other studies demonstrate the importance of exosomal miRNAs as biomarkers of disease.

Influenza virus exposure activates both innate and adaptive immune responses to protect the cells against a virus attack. Most of these immune responses are coordinated by cytokines and chemokines present in the system. However, if there is an imbalance due to excessive recruitment of neutrophils and monocytes to the site of infection, the increase in cytokine expression could exacerbate inflammation in lungs [21]. Earlier studies reported that H5N1 patient serum showed very high levels of interferon gamma inducible protein (IP-10) and monokine induced by IFN γ (MIG/CXCL9) [22], and this was attributed to the severity of the H5N1 pathogenesis [21]. In vitro studies clearly indicated the induction of interleukins IL-6, IL-8, and RANTES on exposure to the influenza A virus [23]. Thus, cytokines and chemokines play an important role in regulating the influenza virus infection efficiency, inflammation and severity of the disease. The purpose of the present study is to understand the circulating serum miRNA profile of influenza A and B patients and the role of miRNA in modulating the influenza-induced cytokines and chemokines in the serum of influenza-infected patients. Here, we explored the correlation between the circulating exosomal miRNAs and cytokines produced during influenza infection and their specificity to influenza A and B patients.

## 2. Results

### 2.1. Demographic of Study Participants

Fifty-four influenza patients and 30 healthy volunteers who were visiting clinics during the influenza seasons from February 2016 to March 2017 were enrolled in this study. The demographic and clinical information for the study participants is given in Table 1. Patients were screened for influenza-like symptoms or tested with a rapid influenza test to confirm the sickness. Rapid influenza diagnostic tests are immunoassays that can identify the presence of influenza A and B viral nucleoprotein antigens in respiratory specimens and display the result in a qualitative way (positive vs. negative). The 54 influenza patients included 28 females and 26 males. Healthy control subjects included 25 females and 5 males. Over half of the control subjects (63%) had received influenza vaccinations, while only 16% of the influenza A patients and 24% of the influenza B patients had been vaccinated. The influenza A and B patients did not show any significant difference in their clinical symptoms.

### 2.2. Differential miRNA Expression in Influenza A and B Patients

**Figure 1:**
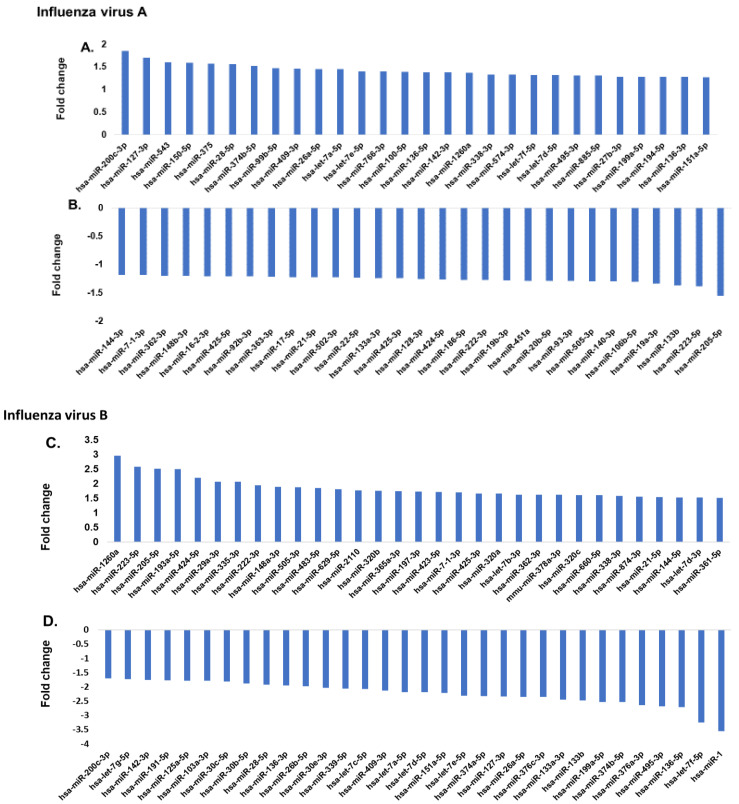
(**A**,**B**) Comparison of serum miRNA expression in influenza A patients. The miRNA levels were determined by qPCR and results presented as the average of 25 patients with a *p* value of 0.05, as compared to the healthy volunteers. The expression level of miRNA from the control group was arbitrarily set at 1. (**C**,**D**) Comparison of serum miRNA expression in influenza B patients. miRNA levels were determined by qPCR and results presented as the average of 29 patients with a *p* value of 0.05, as compared to the healthy volunteers (30 patients). The miRNA was assayed once by qPCR on the MicroRNA Ready-to-Use Serum/Plasma focus panel using the SYBR green master mix. Negative controls, excluding the template from the reverse transcription reaction, were performed. The amplification was performed in a LightCycler^®^ 480 Real-Time PCR System in 384-well plates. The amplification curves were analyzed using the Roche LC software for determination of Cq and the melting curve.

The serum samples collected from the 84 study participants were subjected to serum exosome miRNA array profiling to identify the differentially expressed serum miRNAs. The panel contained 179 miRNAs that are known to be prevalent in human serum samples. Figure 1A shows the most upregulated miRNAs in influenza A patients, while the most downregulated miRNAs are presented in Figure 1B. Figure 1C shows the most upregulated miRNA in influenza B patients’ serum, and Figure 1D shows the most downregulated miRNAs in the influenza B patients as compared to the healthy control subjects. When comparing the control group to the influenza A group using a *t*-test, 26 microRNAs were found to be differentially expressed (*p* < 0.05). Similarly, when comparing the influenza B group to the control group using a *t*-test, 76 microRNAs were found to be differentially expressed at a cutoff of *p* < 0.05. Thirty-one of these differences passed a Benjamini–Hochberg correction at *p* < 0.05. In influenza A patients, hsa-miR-200c (1.9-fold) was the most upregulated miRNA and hsa-miR-205-5p (−1.7-fold) was the most downregulated miRNA. On the other hand, in influenza B patients, hsa-miR-1-3p showed the highest downregulation (−3.54-fold) and hsa-miR-1260a showed the highest upregulation (3.0-fold). Interestingly, neither hsa-miR-1-3p or 1260a showed any significant difference in expression with influenza A patients, while hsa-miR-200c and hsa-miR-205-5p were altered in influenza B patients. Thus, a comprehensive analysis of the miRNA pattern in both strains showed very significant differences in their pattern of expression.

**Figure 2:**
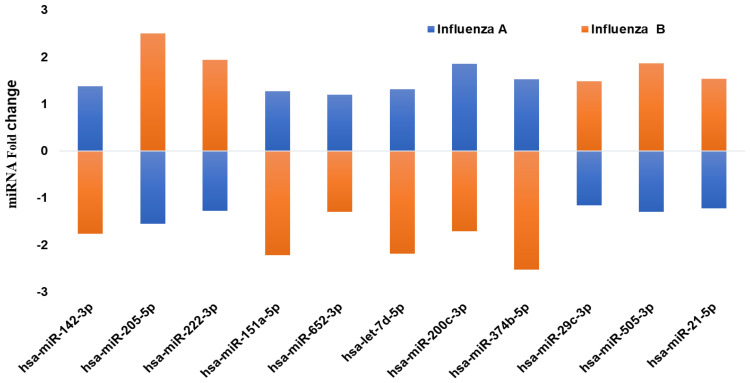
qPCR analysis on the expression of the common miRNAs that are showing a different pattern (up- vs. downregulation) of expression in influenza A and B patients’ serum. The miRNA levels were determined by qPCR and the results presented as the average with a *p* value of 0.05, as compared to the healthy volunteers.

In addition, we also compared some of the common miRNAs that exhibited an opposite effect (down- vs. upregulation) within both influenza A and B patients (Figure 2).

Eleven miRNAs showed a significant difference in expression between the two strains of influenza virus. Interestingly, these 11 miRNAs showed an entirely opposite pattern of expression (up- vs. downregulation) in influenza A and B patients. Hsa-miR-142-3p showed upregulation in influenza A patients (1.4-fold), while it was significantly downregulated in influenza B patients (−1.8-fold). In addition, hsa-miR-205 in influenza A patients exhibited downregulation, but the same miRNA was upregulated in influenza B (2.5-fold). Hsa-miR-374b-5p was downregulated in influenza B, but was 1.2-fold upregulated in influenza A. Similarly, hsa-miR-222-3p showed a downregulation in influenza A, while it was upregulated in influenza B patients. Comparing expression of these 11 miRNAs demonstrates the possibilities of predicting the strains of infection by analyzing these circulating serum miRNAs.

### 2.3. Expression of Cytokines and Chemokines in Patient’s Serum

**Figure 3:**
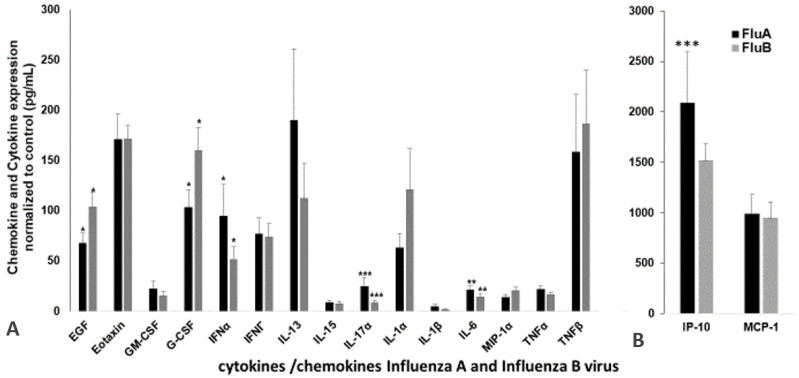
Representative plasma cytokine/chemokine expression levels in the influenza patients (*n* = 54) and controls (*n* = 30) that were significantly different from the control samples. The levels of EGF, eotaxin, GM-CSF, G-CSF, IFNα, IFNγ, IL-13, IL-15, IL-17A, IL-1α, IL-1β, MIP-1β, IL-6, MIP-1α, TNF-α and TNF-β (**A**); IP-10, and MCP-1 (**B**). The *p* values are the difference between A and B are shown with an asterisk(s): * *p* < 0.05; ** *p* < 0.01; *** *p* < 0.001. The commercial Luminex-coated beads and biotinylated antibodies used in this study (mean fluorescence intensity, MFI) were compared between the influenza A and B patients and the healthy controls. Data were analyzed using MILLIPLEX Analyst software. A standard curve for each cytokine and chemokine was generated by mixing known concentrations of the recombinant human cytokines and chemokines. All values were normalized by subtracting the control values of the respective cytokine or chemokine.

To understand whether the influenza virus strain A or B could alter the cytokine or chemokine expression levels, we assessed the levels of 17 chemokines and cytokines that are reported to be influenced in viral infection. Figure 3A shows the difference in expression of both cytokines and chemokines in influenza A and B patients compared to the control group. Several chemokines, such as IP-10, monocyte chemoattractant protein-1 (MCP-1), and MIP-1α, have the potential to recruit and activate leukocytes in response to viral infections. Compared with healthy donors, the serum level of IP-10 was significantly higher (2100 pg/mL) in influenza A patients compared to the influenza B patients (1500 pg/mL) (Figure 3B). MCP-1 did not show any significant difference in expression between the A and B strains, though the levels of expression were approximately 1000 pg/mL higher than in the controls. In addition, influenza A patients showed increased expression of IFNα, GM-CSF, IL-13, IL-17A, IL-1β, IL-6, and TNFα, while influenza B increased the levels of EGF, G-CSF, IL-1α, MIP-1α, and TNF-β. The expression levels of eotaxin were not changed in either influenza A or influenza B patients.

### 2.4. Relationship between Cytokines and Corresponding miRNAs

**Figure 4:**
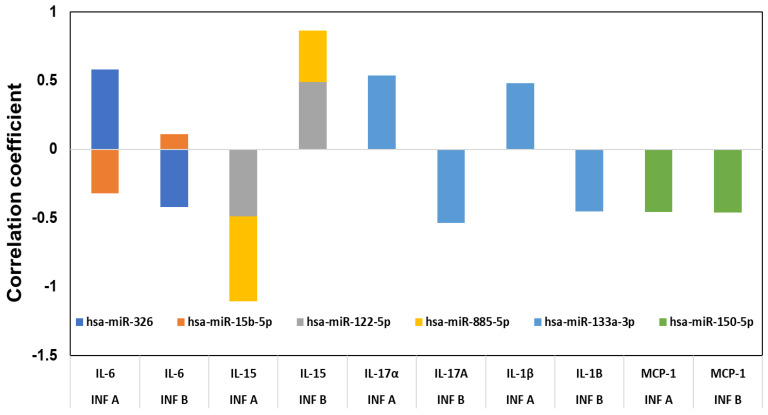
Correlation between the cytokines and chemokines with miRNAs. The cytokine and miRNA levels between the influenza-infected subjects and the controls were compared using *t*-tests, as well as for between influenza A and B, and the false discovery rates calculated (FDR) for the miRNA comparisons. Correlations between various measures were calculated using Pearson’s product–moment correlation coefficient. All comparisons were considered significant at *p* < 0.05.

Next, the associations between miRNAs, chemokines, cytokines, and patient groups were examined by multivariable correlation analysis. The correlations between all the cytokine and chemokine expressions and the expression of the different miRNAs in influenza A and B patients’ serum were assessed (Figure 4). Although we have done a full analysis of all the miRNAs and the 17 cytokines and chemokines in the correlation analysis, only those showing a significant (*p* < 0.05) positive or negative correlation is presented in Figure 4. IL-6 showed a high correlation with hsa-miR-326 and hsa-miR-15b, but with influenza A infection, hsa-miR-326 exhibited a positive correlation, whereas with hsa-miR-15b it showed a negative correlation. With influenza B infection, hsa-miR-326 exhibited a negative correlation, though hsa-miR-15b showed a low positive correlation. With influenza A infection, IL-15 exhibited a high negative correlation with hsa-miR-885 and hsa-miR-122, while hsa-miR-885 and hsa-miR-122 both were positively correlated with influenza B infection. Hsa-miR-133a-3p showed a positive correlation with cytokines IL-17A and IL-1β. In influenza A patients’ serum, hsa-miR-133 had a positive correlation with these cytokines. In contrast, hsa-miR-133a had a negative correlation with IL-17A and IL-1β in influenza B patients. Hsa-miR-150-5p was the only miRNA showing a negative correlation (*p* < 0.05) to MCP-1 in both influenza A and B patients. Both the influenza A and B strains thus show significant differences in their miRNA and cytokine profile.

### 2.5. In Vitro miRNA Analysis

**Figure 5:**
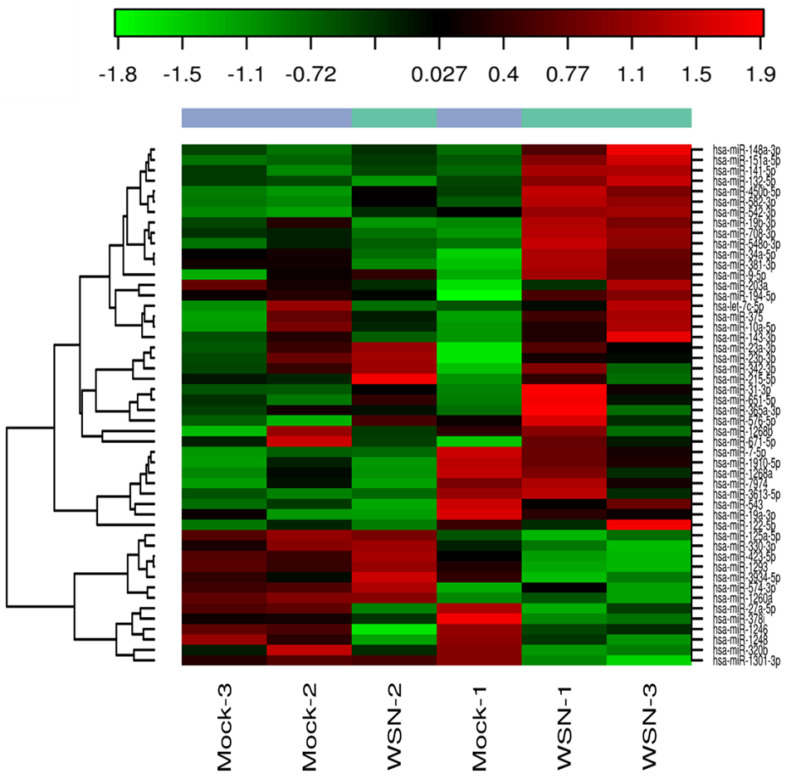
Heat map showing the expression of influenza H1N1-induced miRNAs in human small airway epithelial cells (SAECs). The columns represent the expression pattern of differentially expressed miRNAs on exposure to influenza virus H1N1 and is relative to the mock-infected samples. The red color indicates upregulation and the green color represents the downregulation.

Lung epithelial cells infected with influenza A virus revealed that the early stage infection could significantly alter the miRNA profile. Figure 5 shows the heat map exhibiting the variation in miRNA profile in mock-infected human small airway epithelial cells (SAECs) and human SAECs infected with influenza H1N1. We included only influenza A for the present study because of its wide distribution in most influenza seasons and the strain selected (H1N1/WSN/33) is one of the well-studied IAVs in in vitro experiments and the results can be more easily compared with other published studies. We found several miRNAs were significantly modulated on exposure to influenza A, and the top 20 miRNAs are shown (Table 2) that were significantly altered in human SAECs infected with influenza A (H1N1/WSN). The log fold change along with the *p* values and FDR values are shown. Although all these top 20 miRNAs were not represented in the serum miRNA panel we analyzed, 6 miRNAs were detected (Table 3), showing a similar expression (up- or downregulation) compared to the serum A or B samples. Of these 6 miRNAs, 4 showed upregulation in cells and influenza A patient serum, whereas hsa-miR-324 and hsa-miR-122 was downregulated in cells, similar to the expression pattern in influenza B patient serum.

### 2.6. Functional Analysis

**Figure 6:**
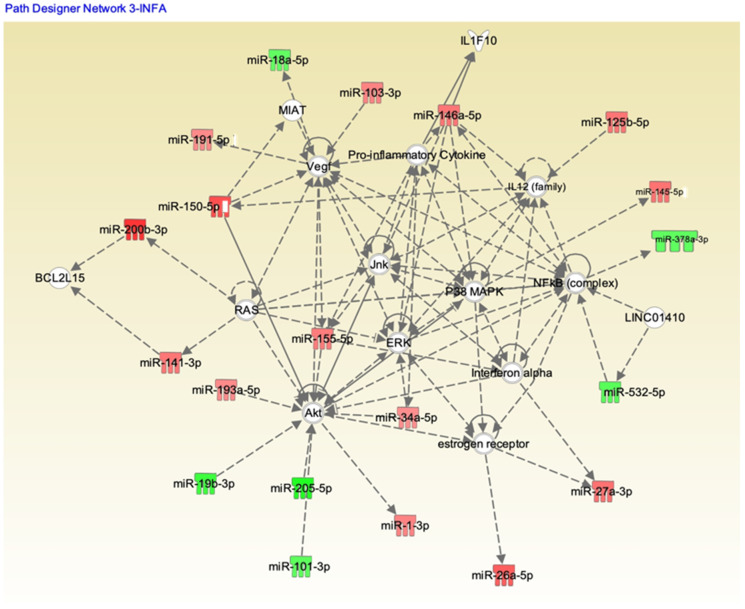
Ingenuity Pathway Analysis (IPA) showing the pathways targeted by different miRNAs in influenza A patients. Upregulation (red) or downregulation (green) of the miRNAs.

**Figure 7:**
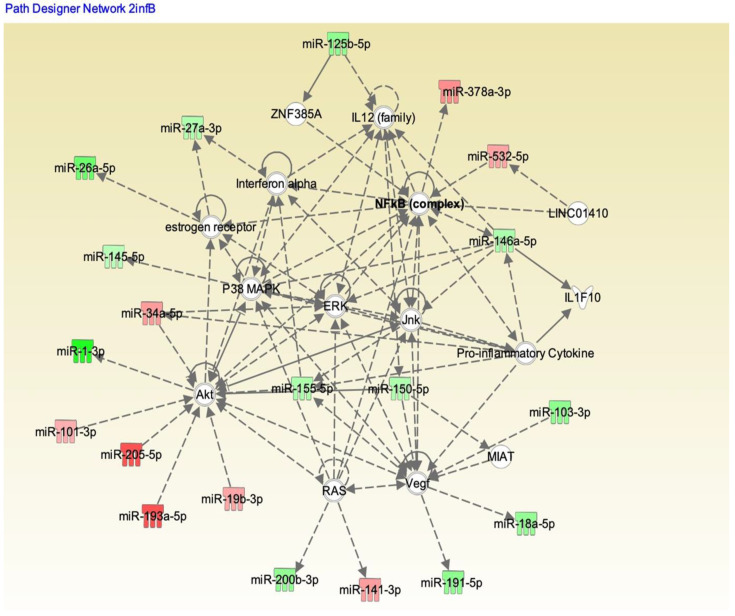
IPA showing the pathways targeted by different miRNAs in influenza B patients. Upregulation (red) or downregulation (green) of the miRNAs.

We analyzed these 179 potential miRNAs using the Ingenuity Pathway Analysis (IPA) “core analysis” to dissect their functional role in influenza A and B patients. Pathway analysis using IPA software showed significant interactions between the miRNAs from the influenza A and B patients’ serum samples (Figure 6 and Figure 7, respectively). Here we identified a number of pathways that are being targeted by the serum miRNA from the influenza A and B patients. Among these pathways, NF-κB signaling, vascular endothelial growth factor (VEGF), AKT, RAS, p38MAPK, ERK, c-Jun N-terminal kinase (JNK), interferon (IFN) α, interleukin-12(IL-12) family, estrogen receptor, and the pro-inflammatory cytokine signaling pathways were the topmost affected pathways in influenza virus infection. In addition to NF-κB (hsa-miR-532), other proteins like IL-12 (hsa-miR125b-5p), IFNα (hsa-miR-27a), and AKT (hsa-miR-1, 205-5p, 101-3p) showed an opposite effect in influenza A compared to influenza B. Other pathway proteins showed a similar effect in both A and B strains, such as RAS (miR-141-3p), VEGF (miR-18a-5p), and ERK (miR-34a-5p). Pathway analysis clearly indicates that the molecular pathway differences in both strains may be attributed to the changes in miRNA expression. The interaction study aided in understanding the role of these essential molecules in influenza A and B patients. The intensity of red indicates upregulation while green indicates downregulation of certain molecules. These data also reflect the interactions of the relevant protein molecules being targeted by multiple miRNAs. For example, the AKT molecule could be targeted by miR-155, miR-193, miR-34, miR-1, miR19, miR-205, and miR101. In addition, a single miRNA may target multiple protein molecules, such as miR-155 targets AKT, IFNα, and VEGF.

## 3. Discussion

The demographic data clearly indicates no significant difference in symptoms was reported for influenza A and B patients. Among the patients recruited for our study, 19 participants in the healthy control group had received an influenza vaccination, whereas only four patients had been vaccinated in the influenza A-positive group and seven patients had been vaccinated in the influenza B-positive group when they were enrolled. This indicates that vaccination may have possibly provided some protection to some of the participants. In this study, a significant difference in the serum miRNA profile was observed in influenza virus A- and B-infected patients. The difference in expression was analyzed by isolating the exosomes from serum samples and quantifying the 179 known miRNAs that circulate in the bloodstream of mice to identify the most important changes that occur after infection of each virus. In recent years, influenza B has surged as one of the leading causes of seasonal infection compared to infections with influenza A [2]. In particular, influenza B infections were more than 50% of the influenza infections in China during the influenza season of 2015/2016 [24]. Although influenza B is less severe in adults, it causes significant problems in children [25].

The serum-circulating miRNA profiles of the influenza A and B patients showed differences in expression, suggesting a significant difference in viral pathologies and pattern of infection. It is difficult to differentiate whether a patient was infected with influenza A or B based on the clinical symptoms. A molecular biomarker has high importance if it can be identified in a sample obtained through a minimally invasive procedure. Thus, in our study, differentially regulated miRNAs and cytokines have significant potential roles as biomarkers for the early detection of disease. miRNA-205-5p, one of the highly expressed miRNAs in influenza B patients, has been shown to be a key regulator of endometrial cancer [26], tumor promotion, and in epithelial–mesenchymal transition [27]. However, its importance in the infectious disease pathway has not been studied. Hsa-miR-374b-5p, which was downregulated in influenza B patients and upregulated influenza A patients, has been reported to have a role in regulating the PI3K/AKT/IRF3 axis in Japanese encephalitis virus (ssRNA virus in the Flaviviridae family)-infected microglial cells [28]. The activation of the PI3K/AKT pathway during viral infection has been correlated with viral replication, viral entry [29] and virus-induced apoptosis [30], suggesting the possibility that influenza strains A and B may also be using a similar pathway. Earlier studies revealed that the expression of hsa-miR-1260 was highly dysregulated in both whole blood and H1N1-infected cells [31]. We also observed a similar increased expression of serum hsa-miR-1260 in influenza A but not in influenza B patients. In vitro studies using NGS also showed that several miRNAs detected in the serum of influenza A patients exhibited very similar patterns of expression in human SAECs, except for hsa-miR-324, which exhibited a downregulation in in vitro studies but was upregulated in influenza A patients.

Immune responses elicited by cytokines and chemokines are considered to be an early sign of defense against influenza infection [32]. Excessive inflammation is considered to be a pathological injury during influenza infection that can disrupt the immunological balance and lead to the release of excessive cytokines and chemokines (called the “cytokine storm”), resulting in organ failure and respiratory distress syndrome [33]. Influenza viruses are known to induce different cytokines, such as IL-6, IL-8, IP-10, MIG, TNF, and RANTES, upon activation of normal T cells, and are expressed and secreted from transformed (A549) bronchial epithelial cells [34,35]. The induction of these cytokines is very closely related to the specific strains of the virus. Chemokines can facilitate anti-immune responses by inducing inflammation, while the overwhelming lung inflammation caused by the presence of chemokines can induce lung injury. The miRNA profiles showed a good correlation with the expression of specific cytokines in our study, highlighting the differences between influenza A and influenza B infections. Influenza virus infections are prone to induce pro-inflammatory cytokines in macrophages, both in human and murine models. The in vitro induction of pro-inflammatory cytokines has also been shown in macrophages infected with H5N1 strains [36]. Very high levels of IP-10 and MIG in patients infected with H5N1 virus also have been reported [37]. We found that the IP-10 levels in influenza A and B patients were significantly higher than levels in healthy donors (Figure 3B). The innate and adaptive immunity and host defense against the invading influenza virus were contributed partly by the influx of specific cytokines [22]. Correlation coefficient analysis between miRNA and cytokines and chemokines showed a good correlation between hsa-miR-326 and hsa-miR-15b to IL-6, hsa-miR-885 and hsa-miR-122 to IL-15, hsa-miR-133a to IL-17 and IL-1β, and hsa-miR-150 to MCP-1. Interestingly, both the influenza A and B strains exhibited opposite correlations with most cytokines and corresponding miRNAs (*p* < 0.05), whereas MCP-1 and hsa-miR-150-5p showed negative correlations with both influenza A and B patients. We did not verify (in vitro nor in vivo) any of these miRNA correlations to the corresponding cytokines. MCP-1, which is produced by pulmonary alveolar epithelial cells, mediates inflammation by regulating the migration and recruitment of monocytes in response to influenza infection [38], suggesting high levels of MCP-1 in both influenza A and B patients’ serum. Hsa-miR-150 found in blood and monocytic cells was packaged into microvesicles that were transported to endothelial cells where it acted on the target gene, c-Myb, a transcription factor that regulate cell proliferation, apoptosis, and tumorigenesis [39]. IL-1β is a vital proinflammatory cytokine that mediates immune functions and participates in the resistance to viral infections during the early stages of infection, but an excessive increase in IL-1β may lead to increased lung injury. Our data also show increased IL-1β levels in influenza A patients compared to influenza B patients, which may help explain their difference in pathogenicity. In addition, IL-1β was targeted by hsa-miR-146a and hsa-miR-155 [40,41], but our correlation studies showed that hsa-miR-133a may also have a role in regulating the expression of IL-1β. In addition, the inflammatory response has been altered during influenza infection, as shown in our pathway analysis (Figure 6), wherein hsa-miR-150-5p and hsa-miR-125 were targeted by the IL-12 family of proteins. Interestingly in our study, the miRNA showing the highest downregulation in influenza A (hsa-miR-205) and influenza B was (hsa-miR-1) targeting the Akt pathway. The correlation between miRNAs and cytokines and chemokines suggest that a combination of two or more miRNAs seems to be specific for a particular strain of virus and could be used as a biomarker for the early detection of influenza A or B specifically. The pathway analysis with both strains (Figure 6 and Figure 7) also shows that the role of these miRNAs is very significant in understanding the molecular behavior and their pathogenicity. It is difficult to predict which components (structure of the virus, host range, mutation rate, etc.) of the two viruses are responsible for the differential expression of miRNA in influenza A and B. For example, the M2 protein of influenza A and B varies significantly in their amino acid sequences and influences the function of the ion channel.

There are some limitations in this study, as the sample size for each group of influenza A and B subjects was small, and thus we were not able to further subgroup subjects with respect to either influenza A or B strains. For example, among the influenza A and B patients as well as the healthy control subjects, there were some that were vaccinated, whereas others were not. We also did not subgroup patients based on their demographic and clinical symptoms.

In conclusion, our data indicate that the influenza strains A and B are different in both their miRNA elicitation profiles and in their levels of serum chemokines and cytokines. Using high-throughput Luminex technology, we found that the serum levels of cytokines and chemokines may be used to identify influenza A from influenza B patients. The cytokine/chemokine profile identified in this study along with miRNAs 326, 15b, 122, 885, 133a, and 150 may be useful as targets for new influenza therapies, although more research is needed to understand the differences in molecular interactions between these miRNAs and cytokines/chemokines in influenza A vs. influenza B patients. Our study also suggests that miRNAs and cytokines/chemokines that correlate with their expression may be useful biomarkers for early detection of infection and may be used to discriminate between influenza A and B infections.

## 4. Materials and Methods

### 4.1. Human Subjects

Eighty-four blood samples and nasopharyngeal swabs were collected from 63 influenza patients (25 influenza A-positive, 29 influenza B-positive) and 30 healthy volunteers (influenza-negative) at the West Virginia University Student Health Service Center (Morgantown, WV, USA) and the Sunnybrook Research Institute (University of Toronto, Toronto, Ontario, Canada) during the 2016–2017 influenza season. The study protocol was approved by the National Institute for Occupational Safety and Health (NIOSH, 14-HELD-05XP) and West Virginia University Institutional Review Boards (IRB), and all participants provided written informed consent. Patients having other complications were excluded from the study. Potential subjects were identified based on a positive rapid influenza test or presentation with influenza-like symptoms, as decided by the consulting physician. Patients were confirmed as infected with Influenza A or B by assessing the RNA extracted from their nasopharyngeal swabs in the quantitative polymerase chain (qPCR) reaction method as described by Lee et al. [14]. Consenting participants were asked to complete a questionnaire and their oral temperature was measured. Participants then contributed 5 mL of blood and two nasal swabs (Copan Diagnostics, Murrieta, CA, USA). Blood samples were left at room temperature for 20 min to allow coagulation, and centrifuged for 10 min at 1500× *g*. All samples were immediately stored on ice and transported to the laboratory for processing at the end of the day. The supernatant serum was collected without disturbing the clot. Serum samples were divided into aliquots and stored at −80 °C.

### 4.2. Blood Serum miRNA Array Study

All the serum sample analyses were conducted by Exiqon Services, Denmark. Samples were shipped to the Exiqon facility. Briefly, exosomes were precipitated using the miRCURY Exosome Isolation Kit—Serum and Plasma. Total RNA was extracted from exosomes using the miRCURY™ RNA isolation kit—biofluids (BF) (Qiagen, Germantown, MD, USA). Exosomes in 200 µL of resuspension buffer per sample were transferred to a microcentrifuge tube and 60 µL of Lysis solution BF containing 16.67 µg/mL of MS2 bacteriophage RNA and RNA spike-in template mixture was added to each sample. The tube was mixed and incubated for 3 min at room temperature, followed by an addition of 20 µL protein precipitation solution BF. The tube was incubated for 1 min at room temperature and centrifuged at 11,000× *g* for 3 min. The clear supernatant was transferred to a new collection tube, and 270 µL isopropanol was added. The solutions were mixed and transferred to a binding column. The column was incubated for 2 min and emptied using a vacuum manifold. A total of 100 µL of wash solution 1 BF was added to the spin column. The liquid was removed using a vacuum manifold, and 700 µL of wash solution 2 BF was added. The liquid was then removed using a vacuum manifold, and dried. The dry spin column was transferred to a new collection tube and 50 µL RNase-free water was added directly onto the membrane of the spin column and centrifuged at 11,000× *g*. The RNA was stored at −80 °C.

### 4.3. miRNA RT-PCR

Seven µL RNA was reverse transcribed in 35 µL reactions using the miRCURY LNA™ Universal RT microRNA PCR using Polyadenylation and cDNA synthesis kit (, Qiagen, Germantown, MD, USA). cDNA was diluted 50× and assayed in 10 μL qPCR reactions according to the protocol for miRCURY LNA™ Universal RT microRNA PCR; each miRNA was assayed once by qPCR on the MicroRNA Ready-to-Use Serum/Plasma focus panel using ExiLENT SYBR Green master mix. Negative controls, excluding the template from the reverse transcription reaction, were performed and profiled like samples. The amplification was performed in a LightCycler^®^ 480 Real-Time PCR System (Roche, Indianapolis, IN, USA) in 384-well plates. The amplification curves were analyzed using the Roche LC software, both for determination of Cq (by the 2nd derivative method) and for the melting curve analysis. The amplification efficiency was calculated using algorithms similar to the LinReg software. All assays were inspected for distinct melting curves and the Tm was checked to be within the known specifications for the assay. In addition, assays with 5 Cqs less than the negative control, and with Cq < 37, were included in the data analysis. Data that did not pass these criteria were omitted from any further analysis. Cq was calculated as the 2nd derivative.

### 4.4. Cells

Human primary small airway epithelial cells (SAEC) were purchased from PromoCell GmbH (Heidelberg, Germany) and sub-cultured in media and supplemented with growth factors recommended by the supplier. Madin Darby Canine Kidney (MDCK) cells were cultured in Eagle’s Minimum Essential Medium (ATCC) supplemented with 10% fetal bovine serum, 100 IU/mL penicillin, and 100 µg/mL streptomycin sulfate. MDCK cells were used for the propagation of influenza virus H1N1 (A/WSN/33). Influenza virus H1N1 (A/WSN/33) was a kind gift from Prof. Robert A. Lamb (Northwestern University, Chicago, IL, USA) and cultivation and maintenance of the virus was carried out as described earlier [4,5,6].

### 4.5. Next-Generation Sequencing (NGS)

NGS analysis was conducted on total RNA isolated from human small airway epithelial cells (SAECs) infected with influenza virus H1N1 (A/WSN/33) to identify differentially expressed host miRNAs. SAECs were infected with influenza virus for 3 h, as described earlier [4] All experiments were repeated three times in duplicates. Total RNA including miRNA was isolated from infected and uninfected cells using the miReasy kit (Qiagen, Rockville, MD, usa) and was sent to Exiqon for NGS analysis. The quality of the RNA was assessed by an Agilent bioanalyzer (Santa Clara, CA, USA).

### 4.6. NGS Data Analysis

The amplification efficiency was calculated using algorithms similar to the LinReg software. All assays were inspected for distinct melting curves and the Tm was checked to be within the known specifications for the assay. Assays were in the range of 5 Cq less than the negative control and with Cq < 37 to be included in the data analysis. Data that did not pass these criteria were omitted from any further analysis. Cq was calculated as the 2nd derivative. Using NormFinder, the best normalizer was found to be the average of the assays detected in all samples. All data were normalized to the average of the assays detected in all samples (average—assay Cq). When comparing the two groups, a few miRNAs were found to be differentially expressed when using a *p*-value cutoff of 0.05. Two of these pass the Benjamini–Hochberg correction for multiple testing at a significance level of 0.05. The data obtained were subjected to statistical analysis and differentially regulated miRNAs in infected and uninfected cells were reported.

### 4.7. Analysis of Cytokines and Chemokines

Cytokines and chemokines were analyzed in 25 µL serum samples from each patient, using multiplex assays according to the manufacturer’s instructions. Seventeen cytokines and chemokines (EGF, eotaxin, GM–CSF, G–CSF, IFN-α, IFN-γ, IL–6, IL–13, IL-15, IL–17A, IL–1β, IL-1α, IP-10, MCP–1, MIP–1α, TNF-α, and TNF-β) (Human cytokine 17-plex panel, Millipore, MA, USA) were quantified using a Luminex 200 instrument (Luminex Corp., Austin, TX, USA). Data were analyzed using MILLIPLEX Analyst software in accordance with the manufacturer’s instructions. A standard curve for each cytokine and chemokine was generated by mixing known concentrations of recombinant human cytokines and chemokines.

### 4.8. Biological Function Analysis

Entire miRNA data of influenza A and B were imported into the Ingenuity Pathway Analysis (IPA) software (Qiagen, Redwood City, CA, USA) for the biological functional analysis. The serum miRNA data of the influenza A and influenza B patients were subjected to IPA analysis, and the resulting pathway networks showed differentially regulated genes and corresponding miRNA that influence the regulation of NF-kβ, Jnk, VEGF, ERK, p38MAPK, and IFN.

### 4.9. Statistical Analysis

All analyses were generated using SAS/STAT software, Version 9.3 of the SAS system for Windows (SAS Institute, Cary, NC, USA). When necessary, data were transformed by calculating the natural log of each value prior to analysis to meet the assumptions of the statistical tests (homogeneity of variance). The cytokine and miRNA levels were compared using *t*-tests between the influenza-infected subjects and controls, and for between influenza A and B; in turn, the false discovery rates (FDR) were calculated for miRNA comparisons. Correlations between various measures were calculated using Pearson’s product–moment correlation coefficient. All comparisons were considered significant at *p* ≤ 0.05.

## Figures and Tables

**Table 1:** Demographic and clinical symptoms of study participants.

	Control		Flu A		Flu B	
Number of subjects	30		25		29	
Gender	Female 25, Male 5		Female 16, Male 9		Female 12, Male 17	
	Mean	SD	Mean	SD	Mean	SD
Age (years)	29.89	6.24	20.8	3.13	21.48	4.83
Height (inches)	65.56	3.56	66.52	3.38	68.17	3.7
Weight (lb)	169.8	60.09	153.44	37.2	165.85	37.14
Temperature (°F)	98.51	0.44	99.54	1.4	99.93	1.13
Number of days of symptoms			2.66	1.88	2.26	1.31
Smoker	1		2		2	
Symptoms reported by subjects						
Fever/chills			22		22	
Headache			21		25	
Fatigue			23		24	
Cough			22		21	
Sore throat			20		21	
Sinus congestion			18		18	
Runny nose			18		23	
Sneezing			11		12	
Muscle aches			20		23	
Received influenza vaccine within last 6 months	19		4		7	

**Table 2:** Top twenty most differentially expressed miRNAs. Data obtained from the next generation sequencing (NGS) analysis of SAEpCs exposed to H1N1 virus as detailed in methods.

miRNAs	logFc	logCPM	*p*-Value	FDR	H1N1 AVG
hsa-mi-375	1.9058	7.6845	6.2031 × 10^−8^	1.8549 × 10^−5^	79.67
hsa-miR-3182	−1.4792	6.0171	1.8421 × 10^−7^	2.7538 × 10^−5^	56.00
hsa-miR-1268b	−1.0368	4.2435	7.5113 × 10^−5^	7.4862 × 10^−5^	15.67
hsa-miR-3613-5p	−0.9121	4.5199	7.9481 × 10^−5^	0.0059	10.33
hsa-miR-1268a	−0.7560	4.1945	0.0003	0.0203	14.00
hsa-miR-215-5p	−0.8795	5.1673	0.0004	0.0202	28.67
hsa-miR-143-3p	0.8751	7.8209	0.0004	0.0213	101.67
hsa-miR-219a-1-3p	−0.7313	4.4770	0.0008	0.0297	17.00
hsa-miR-199a-5p	1.0071	3.0778	0.0011	0.0354	2.67
hsa-miR-199a-3p	1.2813	3.5893	0.0013	0.0395	4.00
hsa-miR-122-5p	−1.2114	6.5600	0.0015	0.0405	84.33
hsa-miR-4791	−0.7608	3.5122	0.0025	0.0620	8.33
hsa-miR-1307-5p	−0.6667	3.5460	0.0038	0.0868	8.33
hsa-miR-135b-5p	−0.4602	7.8092	0.0041	0.0868	164.67
hsa-miR-10a-5p	0.4369	6.2633	0.0246	0.4912	41.33
hsa-miR-4521	0.6325	4.5232	0.0277	0.4912	11
hsa-miR-340-5p	−0.3601	7.838	0.0279	0.4912	163.33
hsa-miR-324-5p	−0.4859	3.2407	0.0325	0.5392	6.33
hsa-miR-27a-5p	0.3404	10.6223	0.0405	0.6379	886.33
hsa-miR-134-5p	0.5735	3.462	0.0451	0.6754	5

Note: log fold change (logFc) between groups H1N1 and Control, log CPM is the log of counts per million, raw *p*-values, Benjamini-Hochberg FDR corrected *p*-value and the average raw values per group are given.

**Table 3:** Comparison of miRNA in small airway epithelial cells treated with H1N1 and serum miRNAs of influenza A and B patients.

	Fold Change		
miRNA	Cells H1N1	Serum Influenza A	Serum Influenza B
hsa-miR-375	1.91	1.575	1.061
hsa-miR-143-3p	0.875	1.083	1.335
hsa-miR-1991-5p	1.007	1.279	−2.526
hsa-miR-199-3p	1.281	1.032	−1.643
hsa-miR-324-5p	−0.486	1.119	−1.541
hsa-miR-122-5p	−1.211	1.153	−1.063

Data compared from the Next generation sequencing analysis (cells) and from serum PCR array, *p* < 0.05.

## Data Availability

Reagents or further information may be obtained from the lead contact Sreekumar Othumpangat (seo8@cdc.gov).
